# Association of Demographic Disparities in Maternal Oral Hygiene Status, Caries Experience, and Oral Health Behavior Toward Children in Chengalpattu District, India: A Cross-Sectional Study

**DOI:** 10.7759/cureus.72989

**Published:** 2024-11-04

**Authors:** Agalya Sivakumar, Jagannatha G Venkatarayappa, Nagaland Tirupati, Cyril H Benedict, Vidhya Gunasekaran, Naziya K Babu

**Affiliations:** 1 Department of Public Health Dentistry, Chettinad Dental College and Research Institute, Chennai, IND

**Keywords:** demographic disparity, maternal-child health, maternal oral health, rural area, urban area

## Abstract

Background and objective: Mothers are the primary caregivers throughout early childhood, playing a vital role in developing their child's oral health behavior, as oral conditions in primary teeth can have a considerable negative impact on the child's growth. This study aims to assess the association between maternal oral hygiene status, caries experience, and oral health behavior (OHB) toward their children.

Methodology: A cross-sectional study was conducted among 226 mothers of children aged nine months to three years visiting the primary health centers (PHCs) in urban (n = 113) and rural (n = 113) areas of Chengalpattu district, India. A 24-item questionnaire assessed and compared their OHB toward child oral health. Maternal oral hygiene status and caries experience were also assessed. Mann-Whitney-U test was used to compare the OHB scores between mothers from rural and urban areas, and Spearman’s rank correlation (rho) was used to find the relationship between OHB with oral hygiene and the caries experience of the participants.

Results: The results showed significant differences in OHB scores between mothers from rural (mean rank: 86.78) and urban (mean rank: 140.22) backgrounds (p <0.001). Urban respondents had significantly higher knowledge (mean rank: 161.09 vs 65.91 for rural) and better oral health practices (mean rank: 132.00 vs 95.00 for rural) compared to their rural counterparts. However, rural respondents significantly showed more positive attitudes than urban respondents (mean rank: 124.80 vs. 102.20 for urban). A strong negative correlation (Spearman's rho of -0.83) was observed between knowledge, attitude, and practice (KAP) scores and Oral Hygiene Index-Simplified (OHI-S) in urban participants, and a moderately negative correlation (-0.683) was observed in rural participants.

Conclusion: Maternal oral hygiene has a significant influence on the OHB toward their children. This study highlights the critical influence of maternal oral health and demographic factors on the OHB of their children, underscoring the need for targeted public health interventions to address these disparities arising out of rural-urban differences and improve the oral health outcomes for both mothers and children. Mothers can thus explicitly play an indispensable role in fostering positive attitudes and healthy habits toward dental health care in children, regardless of the sociodemographic variations.

## Introduction

Child health is a vital asset for any population, with the first year of life being a critical period in a child's development and overall health [1]. Oral well-being is a basic component of general well-being, which plays a vital part in a child’s life [2]. Health behavior encompasses a complex interplay of knowledge, attitudes, and practices that significantly impact oral health [3]. Parents, particularly mothers, serve as key influencers in their children's oral health by modeling good health behaviors. Maternal orientation of dental hygiene and nutritional intake directly affects their children's oral health [4].

Poor maternal oral health can have serious consequences for both the mother and the child's overall health. Children of parents with inadequate oral health are more prone to encounter analogous problems in adulthood than those whose parents uphold good oral health [5]. Dental caries remains the most common chronic disease affecting infants and children, beginning soon after the eruption of primary teeth. The colonization of *Streptococcus mutans* in infants typically occurs around the age of two, with the median age being 26 months referred to as the “window of infectivity.” A child’s level of colonization by cariogenic bacteria is closely linked to the mother’s *S. mutans* level at the time of transmission, indicating a direct relationship between the mother’s oral health and the children [6].

According to the National Oral Health Survey conducted across India, the prevalence of early childhood caries (ECC) ranges from 47.5% to 78.57% in various states [7]. Parental views on oral health are significantly correlated with their children's dental health during early childhood and into later life. To avoid future oral health issues, parents must be aware of their children's oral hygiene and the state of their primary teeth [8]. Oral health in preschool-aged children is mostly determined by behavioral characteristics, such as poor oral hygiene habits, frequent consumption of sugary snacks and drinks, and the absence of preventive dental appointments. The American Academy of Pediatric Dentistry recommends that a dental appointment should occur by the child's first birthday or within six months of the emergence of the first tooth, whichever comes first [9].

Promoting infant oral health through preventive education and dental care can lead to a lifetime free of preventable diseases [10]. Understanding parents' knowledge, attitudes, and practices is important due to the critical role they play in their children's health. It affects both the at-home dental care and their access to dental services. In addition, parental attitudes and presumptions are important for supporting their children's dental health [11].

Educating mothers about infant oral health care can significantly reduce the prevalence of dental caries in children. The American Academy of Pediatric Dentistry recommends assessing mothers’ knowledge and attitudes to develop effective child oral health promotion programs. People living in rural areas often suffer restricted access to prophylactic and therapeutic oral health care services due to the economic, geographical, and cultural barriers that negatively impact oral health outcomes [10].

The first step in any health promotion strategy is to identify areas of concern by evaluating the community’s knowledge, attitudes, and practices regarding health issues. This study aims to assess and compare the oral health behavior (OHB) - knowledge, attitudes, and practices - of mothers of preschool children in both rural and urban primary health centers (PHCs) in Chengalpattu, India, concerning child oral health, and to explore the association between maternal oral hygiene status, their experience with caries, and their children's oral health behaviors.

## Materials and methods

A cross-sectional study was carried out to assess and compare the OHB (KAP) of mothers of nine- to 36-month-old children visiting the PHCs in urban and rural areas between January 2024 to March 2024. The study protocol was developed according to the STROBE (Strengthening the Reporting of Observational Studies in Epidemiology) guidelines. Before the start of the study, ethical clearance (IHEC-I/2352/23) was obtained from the institutional ethical committee, and informed consent was obtained from all the study participants. G*Power software, version 3.1.9.4 (Heinrich-Heine-Universität Düsseldorf, Düsseldorf, Germany), was used to estimate the effect size for the current study (0.53) by obtaining the data from the study done by Mishra et al. [12] and by fixing a 1% level of significance (alpha) and 95% power. The sample size was estimated to be 226 in total, with each group containing 113 participants. The Chengalpattu district comprises 39 PHCs (20 rural PHCs and 19 urban PHCs). One PHC was selected from each category by multistage stratified random selection, following the acquisition of necessary permissions from the authorities, to attain the requisite sample size.

Mothers with children aged nine months to three years who were visiting the PHCs for routine checkups and vaccination schedules and who were willing to participate and give consent were included in the study. Mothers from the medical or paramedical sectors were excluded, as they may have more advanced knowledge of oral hygiene and health due to their training, which could significantly influence their OHB and practices, creating a bias in comparing them with mothers from non-medical backgrounds. Mothers suffering from any systemic/psychological illness were excluded from the study, as it can affect personal health behaviors, including oral hygiene practices, and their inclusion could introduce variability unrelated to the demographic disparities the study aimed to examine. The study participants were divided into two groups - Group A (rural) and Group B (urban) - according to the Rural Urban Classification for Census 2021 in the “Census of India 2021 Circular No. 2.”

Data collection was conducted using a face-to-face interview method, during which a questionnaire was administered, followed by an oral examination. Since the investigator recorded the responses, the questionnaire was kept in English. A customized questionnaire with four domains was used to evaluate maternal OHB toward their children's oral health. The four domains included demographics, knowledge, attitude, and practice, which were measured on a nominal scale.

Section A gathered demographic information such as the mother's age, education, and income; section B consisted of 12 questions to assess maternal knowledge; section C included six questions on maternal attitude, and section D contained six questions regarding the mother's practices and knowledge related to maintaining their child's oral health.

A score of 1 was given for correct responses in sections B, C, and D, and a score of 0 was given for incorrect answers. The total possible score was 24, with knowledge scores ranging from 0 to 12, attitude scores from 0 to 6, and practice scores from 0 to 6. The scoring for the questionnaire was categorized as given in the study conducted by Mishra et al. [12]. Participants who answered more than 16 questions (>70%) correctly were rated as having a "good" knowledge, attitude, and practice (KAP) score. Those answering between nine and 16 questions (37.5%-66%) correctly were rated as "better," while those answering fewer than nine questions (<37.5%) correctly were rated as "poor."

Face and content validation were conducted to ensure the questionnaire's relevance, sequence, and adequacy in measuring the intended variables. The primary investigator held face-to-face interviews with 15 experts, achieving an 88.9% overall agreement. Content validity was assessed using Lawshe’s content validity ratio (CVR), with experts rating each item on a four-point scale. A minimum CVR of 0.49 was required to retain an item, and all 24 questions exceeded this threshold.

The questionnaire was pilot-tested on 40 mothers attending the peripheral center of Chettinad Dental College and Research Institute, Chennai India, to assess its feasibility and suitability for the main study and check its test-retest reliability. These participants were not included in the final study. The test-retest reliability showed strong consistency, with an alpha value greater than 0.83.

An oral examination was conducted to assess the maternal oral hygiene status and caries experience. Oral Hygiene Index-Simplified (OHI-S) [13] was used to assess oral hygiene status and decay-missing-filled teeth (DMFT/S) index [14] for caries experience, which was recorded as continuous variables. A single examiner conducted the examination using the WHO methods (2013), and the clinical examination was carried out at the PHCs using a regular chair, artificial light, mouth mirror, and explorer. The association between maternal oral hygiene status and their caries experience with OHB toward their children was assessed.

For statistical analysis, IBM SPSS Statistics for Windows, version 21 (released 2012; IBM Corp., Armonk, NY) was used after entering the data into a Microsoft Excel sheet (Microsoft Corp., Redmond, WA). For descriptive statistics, frequency of distribution in relation to sociodemographic data was presented. Mann-Whitney U test and Spearman’s correlation test were done, with the level of significance set at 0.05. The comparison of OHB between rural and urban mothers was calculated using the Mann-Whitney U test. Spearman’s correlation was used to find out the correlation between oral health status and caries experience with OHB toward their children. A p-value of ≤0.05 was considered statistically significant and ≤0.001% was highly significant.

## Results

The majority of respondents in both rural and urban areas fall within the 25-35 age group, with 61.9% in rural areas and 72.6% in urban areas. In terms of education, only 29.2% of rural respondents hold a diploma, whereas 84.1% are graduates in the urban population. Additionally, around 61.9% of rural respondents have an annual income below 12,444 INR, and 55.8% of urban respondents have an income between 62,273 and 93,380 INR (Table 1).

**Table 1:** Demographic details

Variables	Rural (n = 113)	Urban (n = 113)	Total (n = 226)
Age (years)			
<25	36 (31.9%)	22 (19.5%)	58 (25.7%)
25-35	70 (61.9%)	82 (72.6%)	152 (67.3%)
>35	7 (6.2%)	9 (8%)	16 (7.1%)
Total	113	113	226
Educational status			
Illiterate	14 (12.4%)	6 (5.3%)	20 (8.8%)
Primary school	9 (8%)	0 (0%)	9 (4%)
Middle school	17 (15%)	3 (2.7%)	20 (8.8%)
High school	27 (23.9%)	0 (0%)	27 (11.9%)
Intermediate/diploma	33 (29.2%)	7 (6.2%)	40 (17.7%)
Graduate	13 (11.5%)	95 (84.1%)	108 (47.8%)
Professional honors	0 (0%)	2 (1.8%)	2 (0.9%)
Total	113	113	226
Income (INR)			
≤₹12,445	70 (61.9%)	12 (10.6%)	82 (36.3%)
₹12,445-₹37,324	27 (23.9%)	0 (0%)	27 (11.9%)
₹37,325-₹62,272	5 (4.4%)	63 (55.8%)	68 (30.1%)
₹62,273-₹93,380	0 (0%)	24 (21.2%)	24 (10.6%)
₹93,381-₹1,24,488	8 (7.1%)	9 (8%)	17 (7.5%)
₹1,24,489-₹2,49,043	3 (2.7%)	5 (4.4%)	8 (3.5%)
>₹2,49,044	0 (0%)	0 (0%)	0 (0%)
Total	113	113	226

A significant portion of urban respondents (47.8%) recognize that a child’s teeth should be cleaned when the first primary tooth erupts, while a substantial percentage of rural respondents (75.2%) delay cleaning until all teeth have erupted. Both rural (85.8%) and urban (86.7%) respondents are aware that sugary foods can cause caries. Awareness of the risks associated with nighttime breastfeeding is low among rural respondents, with only 4.4% acknowledging the risks compared to 70.8% in urban areas. Similarly, only 6.2% of rural respondents recognize the harmful effects of nighttime bottle feeding compared to 65.5% of urban respondents. A vast majority of respondents, both rural (96.5%) and urban (75.2%), are unaware of pit and fissure sealants (Table 2).

**Table 2:** Questions pertaining to knowledge

Questions	Responses	Rural (n = 113)	Urban (n = 113)	Total (n = 226)
When should the cleaning of a child’s teeth begin?	When the first primary tooth erupts	0 (0%)	54 (47.8%)	54 (23.8%)
	When all teeth erupt	85 (75.2%)	53 (46.9%)	138 (61.1%)
	Don’t know	28 (24.8%)	6 (5.3%)	34 (15.1%)
Does sugary food, candies, and pacifiers dipped in sweet liquids cause caries?	Yes	97 (85.8%)	98 (86.7%)	195 (86.3%)
	No	3 (2.6%)	15 (13.3%)	18 (8.0%)
	Don’t know	13 (11.6%)	0 (0%)	13 (5.7%)
Does frequent snacking predispose to dental caries?	Yes	78 (69%)	113 (100%)	191 (84.5%)
	No	5 (4.4%)	0 (0%)	5 (2.2%)
	Don’t know	30 (26.5%)	0 (0%)	30 (13.3%)
When should cariogenic food be given to the child?	Between meals	83 (73.5%)	35 (31%)	118 (52.2%)
	With meals	9 (8.0%)	72 (63.7%)	81 (81%)
	At bedtime	0 (0%)	0 (0%)	0 (0%)
	Don’t know	21 (18.6%)	6 (5.36%)	27 (11.9%)
Can prolonged nocturnal breastfeeding cause dental caries?	Yes	5 (4.4%)	80 (70.8%)	85 (37.6%)
	No	4 (3.5%)	0 (0%)	4 (1.8%)
	Don’t know	104 (92%)	33 (29.2%)	137 (60.6%)
Is nighttime bottle feeding detrimental to children’s oral health?	Yes	7 (6.2%)	74 (65.5%)	81 (35.8%)
	No	2 (1.8%)	3 (2.7%)	5 (2.2%)
	Don’t know	104 (92%)	36 (31.9%)	140 (61.9%)
Can regular cleaning of teeth protect against dental caries?	Yes	74 (65.4%)	108 (95.6%)	182(80.5%)
	No	3 (2.7%)	2 (1.8%)	5 (2.2%)
	Don’t know	36 (31.9%)	3 (2.7%)	39 (17.3%)
Does the child's toothpaste contain fluoride?	Yes	7 (6.2%)	67 (59.3%)	74 (32.7%)
	No	0 (0%)	7 (6.2%)	7 (3.1%)
	Don’t know	106 (93.8%)	39 (34.5%)	145 (64.2%)
Does fluoride toothpaste prevent tooth decay?	Yes	7 (6.2%)	65 (57.5%)	72 (31.9%)
	No	8 (7.1%)	2 (1.8%)	10 (4.4%)
	Don’t know	98 (86.7%)	46 (40.7%)	144 (63.7%)
What is the amount of toothpaste that should be applied for children under the age of 3 years?	Smear size/rice grain	11 (9.7%)	85 (75.2%)	96 (42.4%)
	Peanut size	68 (60.2%)	16 (14.2%)	84 (37.2%)
	The full length of the brush size	18 (15.9%)	0 (0%)	18 (8%)
	Don’t know	16 (14.2%)	12 (10.6%)	28 (12.4%)
Do you know about pit and fissure sealants?	Yes	4 (3.5%)	28 (24.8%)	32 (14.2%)
	No	109 (96.5%)	85 (75.2%)	194 (85.8%)
Do you know that a dental home should be established for the child within six months of eruption of the first tooth and no later than 12 months of age?	Yes	0 (0%)	14 (12.4%)	14 (6.2%)
	No	113 (100%)	99 (87.6%)	212 (93.8%)

Regular dental visits are not common, with only 39.8% of rural respondents and 76.1% of urban respondents acknowledging their importance. While 80.5% of urban respondents agree that mothers should brush their child’s teeth until a certain age, only 27.4% of rural respondents share this understanding (Table 3).

**Table 3:** Questions pertaining to attitude

Questions	Responses	Rural	Urban	Total
Milk teeth are replaced by permanent teeth and do not require brushing.	Agree	31 (27.4%)	11 (9.7%)	42 (18.6%)
	Disagree	70 (61.9%)	99 (87.6%)	169 (74.8%)
	Uncertain	12 (10.6%)	3 (2.7%)	15 (6.6%)
Good oral health positively influences good overall health in an individual.	Agree	68 (60.1%)	101 (89.4%)	169 (74.8%)
	Disagree	3 (2.7%)	0 (0%)	3 (1.3%)
	Uncertain	42 (37.2%)	12 (10.6%)	54 (23.9%)
You feel anxious/ scared about dental consultation for your child.	Agree	58 (51.3%)	97 (85.8%)	155 (68.6%)
	Disagree	3 (2.7%)	6 (5.3%)	9 (4%)
	Uncertain	52 (46%)	10 (8.8%)	62 (27.4%)
The child should be taken for regular visits to the dentist.	Agree	45 (39.8%)	86 (76.1%)	131 (58%)
	Disagree	6 (5.3%)	5 (4.4%)	11 (4.9%)
	Uncertain	62 (54.9%)	22 (19.5%)	84 (37.2%)
Mothers should regularly brush their children’s teeth themselves until a certain age.	Agree	31 (27.4%)	91 (80.5%)	122 (54%)
	Disagree	0 (0%)	6 (5.3%)	6 (2.7%)
	Uncertain	82 (72.6%)	16 (14.2%)	98 (43.4%)
Bacteria implicated in dental caries are transmissible from mother to child.	Agree	5 (4.4%)	45 (39.8%)	50 (22.1%)
	Disagree	0 (0%)	2 (1.8%)	2 (0.9%)
	Uncertain	108 (95.6%)	66 (58.4%)	174 (77%)

Rural respondents are less consistent in brushing habits, with 79.6% brushing only once daily compared to 58.4% in urban areas (Table 4).

**Table 4:** Questions pertaining to practice

Questions	Responses	Rural	Urban	Total
Do you use a pacifier dipped into a sweet liquid for your child?	Yes	3 (2.7%)	0 (0%)	3 (1.3%)
	No	110 (97.3%)	113 (100%)	223 (98.7%)
	Maybe	0 (0%)	0 (0%)	0 (0%)
When did you commence the cleaning of your child’s teeth?	With the eruption of the first tooth	3 (2.7%)	41 (36.3%)	44 (19.5%)
	When more than one tooth erupted	9 (8%)	48 (42.5%)	57 (25.2%)
	When all teeth erupted	101 (89.4%)	24 (21.2%)	125 (55.3%)
How many times does your child brush his/her teeth?	Once in two/three days	21 (18.6%)	5 (4.4%)	26 (11.5%)
	Once daily	90 (79.6%)	66 (58.4%)	156 (68.1%)
	Two times or more	2 (1.8%)	42 (37.2%)	44 (19.5%)
When do you take your child to a dentist?	On regular dental visits as scheduled by the dentist	5 (4.4%)	30 (26.5%)	35 (15.5%)
	When the child suffered toothache	84 (74.3%)	79 (69.9%)	163 (72.1%)
	Have never been to a dentist for the child	24 (21.2%)	4 (3.5%)	28 (12.4%)
What do you do when your child complains of dental pain?	Take him to a dentist	104 (92%)	113 (100%)	217 (96%)
	Give the medicine as prescribed by a nearby medical store/peers/family	9 (8%)	0 (0%)	9 (4%)
If a dentist suggests a treatment to save a painful carious primary tooth, will you get it done?	Yes	97 (85.8%)	97 (85.8%)	194 (85.8%)
	No	16 (14.2%)	16 (14.2%)	32 (14.2%)

The mean knowledge score is significantly higher among urban respondents (mean rank: 161.09) compared to rural respondents (mean rank: 65.91), indicating a substantial knowledge gap. For attitude, rural respondents have a higher mean rank (124.80) than urban respondents (102.20), suggesting that rural populations may hold more positive attitudes but struggle with putting them into practice due to constraints like access to resources. In terms of practice, urban respondents show better habits. This indicates that while attitudes in rural areas might be positive, the application or execution of good dental practices is limited. The overall KAP scores show urban respondents significantly outperforming rural ones (mean rank: 140.22 vs. 86.78). This comprehensive difference reflects both better awareness and better practices in urban settings (Table 5).

**Table 5:** Comparison of OHB (KAP) scores between rural and urban areas Mann-Whitney U test was used. *p ≤ 0.001 indicates high statistical significance. OHB: Oral health behavior; KAP: Knowledge, attitude, and practice.

Variables	Group	N	Mean rank	Sum of ranks	Z	p-value
Knowledge (K)	Rural	113	65.91	7447.50	-11.097	0.001*
	Urban	113	161.09	18203.50		
Attitude (A)	Rural	113	124.80	14102.50	-2.838	0.001*
	Urban	113	102.20	11548.50		
Practice (P)	Rural	113	95.00	9806.50	-4.603	0.001*
	Urban	113	132.00	15844.50		
OHB (KAP)	Rural	113	86.78	9806.50	-6.211	0.001*
	Urban	113	140.22	15844.50		

A strong negative correlation (Spearman's rho) between KAP scores and OHI-S in urban populations (-0.835) and a moderately negative correlation in rural populations (-0.683) were observed (Table 6). This implies that as knowledge, attitude, and practices improve, oral hygiene status also improves, leading to better dental health outcomes.

**Table 6:** Correlation between OHB (KAP) scores and oral hygiene scores Spearman’s correlation was used. *p ≤ 0.001 indicates high statistical significance. OHB: Oral health behavior; KAP: Knowledge, attitude, and practice; OHI-S: Oral Hygiene Index-Simplified.

Variable	Group	OHI-S	
		rho	p-value
Oral health behavior (KAP)	Rural	-0.683	0.001*
	Urban	-0.835	0.001*

However, there is a weak correlation between KAP scores, and DMFT is weaker, especially in rural areas (-0.50) compared to urban areas (-0.030), as shown in Table 7.

**Table 7:** Correlation between OHB (KAP) scores and caries experience scores Spearman’s correlation was used. *p ≤ 0.001 indicates high statistical significance. OHB: Oral health behavior; KAP: Knowledge, attitude, and practice; DMFT: Decay-missing-filled teeth.

Variable	Group	DMFT	
		rho	p-value
Oral health behavior (KAP)	Rural	-0.50	0.001*
	Urban	-0.030	0.001*

## Discussion

The oral health of children is closely linked to the OHB of their mothers or guardians, as oral hygiene and dietary habits are typically formed during infancy and carried into early childhood. Parents, particularly mothers, serve as role models for their children. To our knowledge, there are limited Indian studies that focus on the relationship between maternal oral hygiene, caries experience, and OHB in children in the age group of nine months to three years. The questionnaires specifically examine mothers’ understanding of key risk and protective factors that could impact the oral health of the children and how sociodemographic factors influence this knowledge.

Higher-educated mothers scored higher on KAP compared to lower-educated mothers, which is consistent with studies by Mishra et al. [12] and Ashkanani et al. [15] study. However, this contrasts with the findings from a study conducted in Nigeria by Folayan et al. [16] where maternal socioeconomic factors and decision-making abilities were not associated with the prevalence of ECC. The majority of mothers (61.1%) in the current study agreed that their children should start brushing their teeth after all primary teeth have erupted. This finding aligns with the results of Suresh et al. [17], although it was less so than those reported by Shivaprakash et al. [18].

According to a study by Sowmya et al. [19], 86.3% of mothers believed that sugary foods, candies, and pacifiers dipped in sweet liquids contribute to caries, and 84.5% thought frequent snacking increases the risk of dental caries, which aligns with the findings of our study. The systematic use of fluoride, especially in drinking water, toothpaste, and other dental products, significantly reduces the prevalence of dental caries [10], but only 31.9% of parents were aware that fluoride helps prevent tooth decay. A constantly low understanding of fluoride's role in preventing caries was observed among them. These findings were consistent with earlier research conducted by Sowmya et al. [19], Suresh et al. [17] and Shivaprakash et al. [18].

Relative to the results of Nagarajappa et al. [11] and Priya et al. [20], fewer mothers (nearly 22.1%) agreed that bacteria passed from mother to child can cause tooth decay. However, a significant percentage (96%) of parents across both rural and urban areas reported that they would take their children to a dentist if they experienced tooth pain. The results of this investigation supported the findings of Mehta et al. [21] and Suresh et al. [22], who stated that the primary reason Indians seek dental healthcare services is to relieve pain.

A strong negative correlation was observed between KAP scores and OHI-S scores in both rural and urban populations. However, the correlation between KAP scores and DMFT was weaker, especially in rural areas, while urban areas had a moderate negative correlation, which aligns with Shivaprakash et al. [18]. This suggests that while improved KAP can lead to better oral hygiene, the reduction in dental caries (DMFT) is more complex and may involve other factors such as fluoride access, regular dental visits, and socioeconomic conditions.

Limitations of the study

Data collected through interviews may be subject to social desirability or recall bias. The cross-sectional design limits the determination of causality. As the samples were drawn from a single rural and urban PHCs, caution should be exercised when generalizing the findings.

Recommendations

Mothers in both urban and rural areas should receive dental health advice during prenatal and postnatal care visits. Additionally, comprehensive community-focused oral health promotion programs should be included in public health campaigns managed by governments. These efforts could lessen the prevalence of oral health disorders and assist rural and less educated populations in establishing preventive dental health practices for young children. Further studies should be conducted in large populations, and their implications can be ascertained using longitudinal studies. Parental caregiving behavior, parental anxiety, and parental caries experience should also be assessed along with the caries experience of children.

## Conclusions

Maternal oral hygiene has a significant influence on the OHB toward their children. This study highlights the critical influence of maternal oral health and demographic factors on children's OHB, underscoring the need for targeted public health interventions to address these disparities arising out of rural-urban differences and improve oral health outcomes for both mothers and children. Mothers can thus explicitly play an indispensable role in fostering positive attitudes and healthy habits toward dental health care in children, regardless of the sociodemographic variations.

## Appendices

Study questionnaire

Section A: Demographic Details

1. Age of the mother (years)

a. <25

b. 25-35

c. >35

2. Education of the mother

a. Illiterate

b. Up to middle school

c. Up to senior secondary school

d. Graduate and above

3. Monthly family income (INR)

a. ≥2,49,044

b. 1,24,489-2,49,043

c. 93,381-1,24,488

d. 62,273-93,380

e. 37,325-62,272

f. 12,445-37,324

g. ≤12,444

4. Location of the residential area

a. Rural

b. Urban

Section B: Questions Pertaining to Knowledge

1. When should the cleaning of a child’s teeth begin?

a. When the first primary tooth erupts

b. When all teeth erupt

c. Don’t know

2. Does sugary food, candies, and pacifiers dipped in sweet liquids cause caries?

a. Yes

b. No

c. Don’t know

3. Does frequent snacking predispose to dental caries?

a. Yes

b. No

c. Don’t know

4. When should cariogenic food be given to the child?

a. Between meals

b. With meals

c. At bedtime

d. Don’t know

5. Can prolonged nocturnal breastfeeding cause dental caries?

a. Yes

b. No

c. Don’t know

6. Is nighttime bottle feeding detrimental to children’s oral health?

a. Yes

b. No

c. Don’t know

7. Can regular cleaning of teeth protect against dental caries?

a. Yes

b. No

c. Don’t know

8. Does the child's toothpaste contain fluoride?

a. Yes

b. No

c. Don’t know

9. Does fluoride toothpaste prevent tooth decay?

a. Yes

b. No

c. Don’t know

10. What is the amount of toothpaste that should be applied for children under the age of three years?

a. Smear size/rice grain

b. Peanut size

c. Full length of the brush size

d. Don’t know

11. Do you know about pit and fissure sealants?

a. Yes

b. No

12. Do you know that a dental home should be established for the child within six months of eruption of the first tooth and no later than 12 months of age?

a. Yes

b. No

Section C: Questions Pertaining to Attitude

1. Milk teeth are replaced by permanent teeth and do not require brushing.

a. Agree

b. Disagree

c. Uncertain

2. Good oral health positively influences good overall health in an individual.

a. Agree

b. Disagree

c. Uncertain

3. You feel anxious/scared about dental consultation for your child.

a. Agree

b. Disagree

c. Uncertain

4. The child should be taken for regular visits to the dentist.

a. Agree

b. Disagree

c. Uncertain

5. Mothers should regularly brush their child’s teeth themselves until a certain age.

a. Agree

b. Disagree

c. Uncertain

6. Bacteria implicated in dental caries are transmissible from mother to child.

a. Agree

b. Disagree

c. Uncertain

Section D: Questions Pertaining to Practice

1. Do you use a pacifier dipped into a sweet liquid for your child?

a. Yes

b. No

c. Maybe

2. When did you commence the cleaning of your child’s teeth?

a. With the eruption of the first tooth

b. When more than one tooth erupted

c. When all teeth erupted

3. How many times does your child brush his/her teeth?

a. Once in two/three days

b. Once daily

c. Two times or more

4. When do you take your child to a dentist?

a. On regular dental visits as scheduled by the dentist

b. When the child suffered toothache

c. Have never been to a dentist for the child

5. What do you do when your child complains of dental pain?

a. Take him/her to a dentist

b. Give the medicines as prescribed by nearby medical stores/peers/family

6. If a dentist suggests a treatment to save a painful carious primary tooth, will you get it done?

a. Yes

b. No
